# Attitude Stabilization Control of Autonomous Underwater Vehicle Based on Decoupling Algorithm and PSO-ADRC

**DOI:** 10.3389/fbioe.2022.843020

**Published:** 2022-02-28

**Authors:** Xiong Wu, Du Jiang, Juntong Yun, Xin Liu, Ying Sun, Bo Tao, Xiliang Tong, Manman Xu, Jianyi Kong, Ying Liu, Guojun Zhao, Zifan Fang

**Affiliations:** ^1^ Key Laboratory of Metallurgical Equipment and Control Technology of Ministry of Education, Wuhan University of Science and Technology, Wuhan, China; ^2^ Research Center for Biomimetic Robot and Intelligent Measurement and Control, Wuhan University of Science and Technology, Wuhan, China; ^3^ Hubei Key Laboratory of Mechanical Transmission and Manufacturing Engineering, Wuhan University of Science and Technology, Wuhan, China; ^4^ Precision Manufacturing Research Institute, Wuhan University of Science and Technology, Wuhan, China; ^5^ Hubei Key Laboratory of Hydroelectric Machinery Design & Maintenance, China Three Gorges University, Yichang, China

**Keywords:** AUV (autonomous underwater vehicle), ADRC (active disturbance rejection control), PSO (partial swarm optimization), attitude control, anti-disturbance control

## Abstract

Autonomous Underwater Vehicle are widely used in industries, such as marine resource exploitation and fish farming, but they are often subject to a large amount of interference which cause poor control stability, while performing their tasks. A decoupling control algorithm is proposed and A single control volume–single attitude angle model is constructed for the problem of severe coupling in the control system of attitude of six degrees of freedom Autonomous Underwater Vehicle. Aiming at the problem of complex Active Disturbance Rejection Control (ADRC) adjustment relying on manual experience, the PSO-ADRC algorithm is proposed to realize the automatic adjustment of its parameters, which improves the anti-interference ability and control accuracy of Autonomous Underwater Vehicle in dynamic environment. The anti-interference ability and control accuracy of the method were verified through experiments.

## Introduction

Autonomous Underwater Vehicle (AUV) can adapt excellently to the highly variable and dangerous deep-sea environment and are often used as an important platform for ocean work and underwater inspection. Nowadays, AUV are widely used (Palomeras et al., 2018; Peukert et al., 2018; Chen et al., 2021a) in the fields of infrastructure inspection (Ridao et al., 2010), marine geology (Escartín et al., 2008), underwater archaeology (Bingham et al., 2010), and target search (Cao et al., 2016; Weng et al., 2021). More applications can be realized through the combination of related technologies and AUVs (Sun et al., 2018; Chen et al., 2021b; Hao et al., 2021.; Yang et al., 2021; Zhang et al., 2022). The control of AUV is highly nonlinear (Jia et al., 2012; Roy et al., 2013; Miao et al., 2015; Yun et al., 2022a), with severe coupling of motions between different directions (Tang et al., 2012), while the underwater environment is complex with uncertainties such as waves, water plants, and other disturbances. Therefore, it is significant to carry out the study of control strategies.

For a six-degree-of-freedom AUV, an anti-interference decoupling control algorithm is proposed in this paper in order to solve the problems of its serious attitude coupling and complex and diverse interference in the working environment, and the main contributions of this paper are as follows:

1) A decoupling method is proposed for the six-degree-of-freedom AUV, which solves the problem of serious coupling between variables and realizes that one output variable corresponds to one input variable of the attitude angle.
2) The disturbances are observed and compensated by the ADRC controller, so as to improve the interference resistance of the AUV, and achieves stable control of the attitude of the AUV.
3) The self-tuning of ADRC parameters is achieved with the ADRC algorithm optimized by PSO.

The other parts of this paper are as follows, *Related Work* offers a review of control methods for AUV, *AUV Attitude Anti-Disturbance Decoupling Control* analyzes the forces on the AUV and establishes a mathematical model, and proposes a decoupling algorithm for the problem of severe model coupling, and also improves a Active Disturbance Rejection Control for attitude control of AUV based on Particle Swarm Optimization, *Simulation Experiments* gives a simulation control test of different controllers for environmental disturbances and system changes. and the conclusions of this paper are summarized in *Conclusion*.

## Related Work

There are some control algorithms currently used in AUV, such as PID control, fuzzy control, sliding mode variable structure control, adaptive control, and control strategies that combine multiple control methods.

1) PID control. It is one of the most commonly used control methods in industry today (Kim et al., 2015; Huang et al., 2019; Yu et al., 2019; Xu et al., 2022). The simplicity of the design, the ease of debugging, and no modeling required are the advantages of PID control. However, it is a linear combination of proportional, differential, and integral that simply applies the “deviation between the desired value and the system output value”, which can cause significant overshoot and oscillation when controlling AUV in a strong time-varying environment.
2) Fuzzy control. It accomplishes the control laws by expressing the characteristics of the object model and the control behavior (Jiang et al., 2019a; Liu et al., 2021a; Chen et al., 2021c; Cheng et al., 2021). Khodayari (Khodayari et al., 2015) designed a fuzzy control algorithm combined with an adaptive approach, which was applied to both depth and heading channels of the AUV with good results. Hammad (Hammad et al., 2017) designed a self-tuning fuzzy PID controller to control a multi-input and multi-output fully driven AUV, which is faster and more stable compared to PID accordingly. However, the fuzzy controller needs to adjust a large number of fuzzy variables and related parameters, if the dimensionality of the robot is increased,
3) Sliding mode variable structure control. It uses a sliding surface to improve the control quality of the controller, it will make adjustments according to the state of the system, so that the system follows the set trajectory movement, which enables the system to have good robustness to external disturbances and parameter disturbances (Gao et al., 2013; He et al., 2019; Huang et al., 2020; Li et al., 2020). It is more suitable to control the motion of AUV, but due to the influence of factors such as inertia and time delay, the system is prone to jitter and vibration. Luis (Luis et al., 2016) implemented a sliding-mode variable structure regulation based on the dynamics model of the AUV, which allowed the system to have the desired control effect and to achieve path tracking on the AUV. The high-order sliding mode controller designed by Ruiz-Duarte (Ruiz-Duarte et al., 2015) has theoretically demonstrated good robustness in the longitudinal motion of the AUV and fast response to disturbances. However, there are some engineering difficulties in applying this complex control strategy to practical applications.
4) Adaptive control. This control method is a real-time measurement of the control quantity, and then adjusts its parameters and construction method to achieve the effect of weakening the influence caused by disturbances (Sun et al., 2020a; Liao et al., 2020; Jiang et al., 2021a; Wen et al., 2021). Adaptive control can achieve optimal or suboptimal control after obtaining the mathematical model of the control object. Rath B N (Rath et al., 2020) proposed an adaptive controller with a time delay estimator that successfully predicted the state of the AUV. An accurate model of the control object is obtained and the control effect is very extremely good. F. Rezazadegan (Rezazadegan et al., 2015) used the Lyapunov theory for adaptive rectification of inverse control, which is used for the estimation of uncertain parameters for the tracking control of AUV motion trajectories, and the simulation results show that the control algorithm has some stability. However, the algorithm is based on linear control theory and the transition relies on an accurate mathematical model, therefore the AUV has complex and variable disturbances and time-varying parameters, making it difficult to achieve the desired control effect.

By the above introduction, the current control methods (Caffaz et al., 2010; Xiang et al., 2015; Mcewen et al., 2017; Sun et al., 2020b) can be presumably divided into two types, one uses errors to eliminate errors, which is represented by PID (Duan et al., 2021; Tao et al., 2022a; Yun et al., 2022b; Sun et al., 2022), this method, is independent of the model, adjusts only for the control process without considering the structure and state of the system and has been used in a large number of applications in practical engineering. Another one is the modern control theory represented by sliding mode control and adaptive control (Feng et al., 2017; Li B. et al., 2019; Jiang et al., 2019b; Liu et al., 2022a), these methods rely on the mathematical model of the control system, but by the error with the manufacturing and processing, we are difficult to establish an accurate mathematical model, its external interference and system parameter changes are even more difficult to predict for the AUV operating in an unknown environment, so use these methods in the engineering practice is difficult.

The Active Disturbance Rejection Control (ADRC) algorithm studied in this paper (Han, 2002; Ma et al., 2020; Sun et al., 2021; Tao et al., 2022b), which inherits the characteristics of the PID algorithm, does not depend on the accurate model of the control object, while using modern signal processing techniques to improve its control process, unifies external disturbances and system errors as total disturbances, and then estimates and compensates them, which has the advantage of being easy to use in engineering and at the same time has a strong anti-disturbance capability. Depending on the Particle Swarm Optimization (PSO) (Wang et al., 2013; Liu X. et al., 2021; Bai et al., 2022; Liu et al., 2022b) which is characterized by fast convergence and high computational efficiency (Li et al., 2019b; Jiang et al., 2021b; Liu et al., 2021c; Huang et al., 2021.), Optimizing the parameters of ADRC using PSO can achieve better control effect. In this paper, this control method will be used to solve the problems of nonlinear coupling, external perturbations, and internal parameter variations of the AUV.

## AUV Attitude Anti-Disturbance Decoupling Control

### AUV Mathematical Model

**FIGURE 1:**
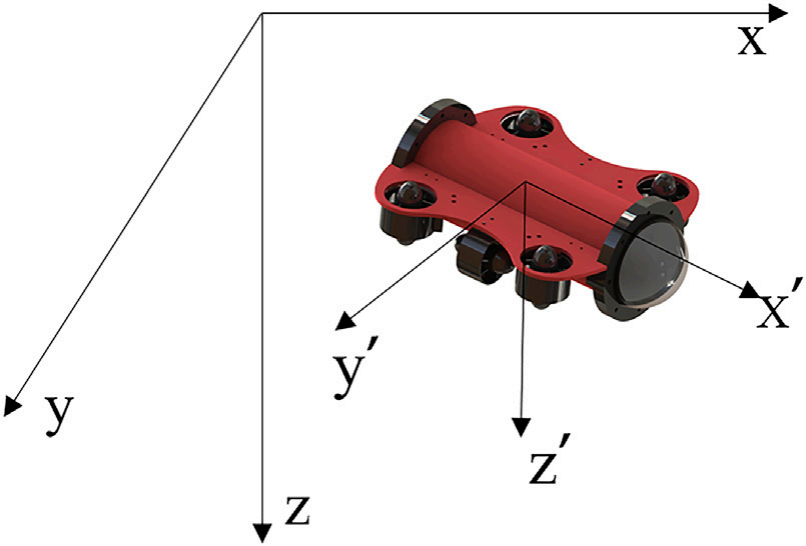
Relationship between ground coordinates and airframe coordinates.

The Active Disturbance Rejection Control technique has the characteristic of not relying on the specific model of the controlled object, but building a relatively accurate model can make our simulation more convincing and more reference value The research in this paper is centered on the AUV which is shown in Figure 1. In this section, the dynamics of AUV shown in Figure 1 is modeled. To simplify the task of model building and subsequent control, the following assumptions are made:

1) Assume that the AUV is a rigid body and its mass does not vary with time.
2) The effect of Earth’s rotation on the motion of AUV is not considered.
3) The flow field in the model is a steady-state flow field and the surrounding water is assumed to be stationary.

In this paper, the thruster distribution of the AUV is used as shown in Figure 1. The motion of the AUV is described using the ground and airframe coordinate systems shown in Figure.

The conversion matrix from the AUV airframe coordinate system to the ground coordinate system is (Li et al., 2019c; Tan et al., 2020; Xiao et al., 2021; Liu et al., 2022c)

$$R^{BE}=\left[\begin{array}{ccc} C_{\theta }C_{\psi } & -C_{\varphi }S_{\psi }+S_{\varphi }S_{\theta }C_{\psi } & {\text{S}}_{\varphi }C_{\psi }+C_{\varphi }S_{\theta }C_{\psi }\\ C_{\theta }S_{\psi } & C_{\varphi }C_{\psi }+S_{\varphi }S_{\theta }C_{\psi } & -{\text{S}}_{\varphi }C_{\psi }+C_{\varphi }S_{\theta }S_{\psi }\\ -S_{\theta } & C_{\theta }S_{\varphi } & C_{\theta }C_{\varphi } \end{array}\right]$$ (1)

The force analysis of AUV has:

$$F_{G}=\left[\begin{array}{l} 0\\ 0\\ mg \end{array}\right],F_{B}=\left[\begin{array}{l} 0\\ 0\\ F_{b} \end{array}\right],F_{A}=\left[\begin{array}{l} F_{x}\\ F_{y}\\ F_{z} \end{array}\right]=\left[\begin{array}{c} F_{5}+F_{6}\\ 0\\ F_{1}+F_{2}+F_{3}+F_{4} \end{array}\right]$$ (2)

Where $F_{G}$ denotes gravity, $F_{B}$ denotes buoyancy, $F_{A}$ denotes the thrust provided by the AUV thrusters, and $F_{A}$ is relative to the airframe coordinate system, so it needs to be converted:

$$F_{\Omega }=R_{A-E}F_{A}=\left[\begin{array}{l} F_{x}C_{\theta }C_{\psi }+F_{z}S_{\varphi }C_{\psi }+F_{z}C_{\varphi }S_{\theta }C_{\psi }\\ F_{x}C_{\theta }S_{\psi }+F_{z}S_{\varphi }C_{\psi }+F_{z}C_{\varphi }S_{\theta }S_{\psi }\\ -F_{x}S_{\theta }+F_{z}C_{\theta }C_{\psi } \end{array}\right]$$ (3)

According to Newton’s Second Law of Motion:

$$\sum F=F_{\Omega }+F_{B}-F_{G}=m\left[\begin{array}{l} \overset{..}{x}\\ \overset{..}{y}\\ \overset{..}{z} \end{array}\right]$$ (4)

Then

$$\left[\begin{array}{l} \overset{..}{x}\\ \overset{..}{y}\\ \overset{..}{z} \end{array}\right]=\frac{1}{m}\left[\begin{array}{l} F_{x}C_{\theta }C_{\psi }+F_{z}S_{\varphi }C_{\psi }+F_{z}C_{\varphi }S_{\theta }C_{\psi }\\ F_{x}C_{\theta }S_{\psi }+F_{z}S_{\varphi }C_{\psi }+F_{z}C_{\varphi }S_{\theta }S_{\psi }\\ F_{b}-mg-F_{x}S_{\theta }+F_{z}C_{\theta }C_{\psi } \end{array}\right]$$ (5)

The angular motion of the AUV is controlled by the moments it is subjected to. The moments to which the AUV is subjected are mainly the moments generated by the thruster thrust and the thruster counter-torque, and the total moment can be expressed as

$$\left[\begin{array}{l} M_{x}\\ M_{y}\\ M_{z} \end{array}\right]=\left[\begin{array}{l} F_{1}X+F_{2}X-F_{3}X-F_{4}X+M_{6}-M_{5}\\ F_{1}Y-F_{2}Y-F_{3}Y+F_{4}Y\\ M_{1}-M_{2}+M_{3}-M_{4}+F_{6}Y-F_{5}Y \end{array}\right]$$ (6)

$F_{i}$ is the thrust generated by the *i*th thruster, X, Y denotes the distance from the thruster to the corresponding coordinate axis, and $M_{i}$ denotes the counter torque generated by the *i*th thruster.

The reaction moment generated by the thrusters can be mostly offset by the forward and reverse propeller settings, and itis much smaller than the moment generated by the thrusters, in order to simplify the model, the total moment can be expressed as:

$$\left[\begin{array}{l} M_{x}\\ M_{y}\\ M_{z} \end{array}\right]=\left[\begin{array}{c} F_{1}X+F_{2}X-F_{3}X-F_{4}X\\ F_{1}Y-F_{2}Y-F_{3}Y+F_{4}Y\\ F_{6}Y-F_{5}Y \end{array}\right]$$ (7)

According to the Newtonian Euler equation:

$$\sum M=\left[\begin{array}{l} M_{x}\\ M_{y}\\ M_{z} \end{array}\right]=Iw-w\times \left(Iw\right)$$ (8)

Among them $w=\left[\begin{array}{l} \dot{\varphi }\\ \dot{\theta }\\ \dot{\psi } \end{array}\right]$

Obtains

$$\sum M=\left[\begin{array}{l} M_{x}\\ M_{y}\\ M_{z} \end{array}\right]=\left[\begin{array}{c} I_{x}\overset{..}{\varphi }+\dot{\theta }\dot{\varphi }\left(I_{y}-I_{z}\right)\\ I_{y}\overset{..}{\theta }+\dot{\varphi }\dot{\psi }\left(I_{z}-I_{x}\right)\\ I_{z}\overset{..}{\psi } \end{array}\right]$$ (9)

The equation of rotation is then obtained as

$$\left[\begin{array}{l} \overset{..}{\varphi }\\ \overset{..}{\theta }\\ \overset{..}{\psi } \end{array}\right]=\left[\begin{array}{c} \frac{I_{y}-I_{z}}{I_{x}}\dot{\theta }\dot{\psi }+\frac{M_{x}}{I_{x}}\\ \frac{I_{z}-I_{x}}{I_{y}}\dot{\varphi }\dot{\psi }+\frac{M_{y}}{I_{y}}\\ \frac{M_{z}}{I_{z}} \end{array}\right]$$ (10)

From Eqs 5, 7, 10, the mathematical model of robot posture control can be obtained:

$$\left[\begin{array}{l} \overset{..}{x}\\ \overset{..}{y}\\ \overset{..}{z}\\ \overset{..}{\varphi }\\ \overset{..}{\theta }\\ \overset{..}{\psi } \end{array}\right]=\left[\begin{array}{c} \frac{1}{m}\left(F_{x}C_{\theta }C_{\psi }+F_{z}S_{\varphi }C_{\psi }+F_{z}C_{\varphi }S_{\theta }C_{\psi }\right)\\ \frac{1}{m}\left(F_{x}C_{\theta }S_{\psi }+F_{z}S_{\varphi }C_{\psi }+F_{z}C_{\varphi }S_{\theta }S_{\psi }\right)\\ \frac{1}{m}\left(F_{b}-mg-F_{x}S_{\theta }+F_{z}C_{\theta }C_{\psi }\right)\\ \frac{I_{y}-I_{z}}{I_{x}}\dot{\theta }\dot{\psi }+\frac{F_{1}X+F_{2}X-F_{3}X-F_{4}X}{I_{x}}\\ \frac{I_{z}-I_{x}}{I_{y}}\dot{\varphi }\dot{\psi }+\frac{F_{1}Y-F_{2}Y-F_{3}Y+F_{4}Y}{I_{y}}\\ \frac{F_{6}Y-F_{5}Y}{I_{z}} \end{array}\right]$$ (11)

$F_{i}$ denotes the thrust of the *i*th motor. Table 1 is the relevant parameters of AUV.

**TABLE 1:** Related parameters of AUV.

M (kg)	G (m/s^2^)	X (m)	Y (m)	I_x_ (kg•m^2^)	I_y_ (kg•m2)	I_z_ (kg•m2)
5.8	9.8	0.17	0.2	0.078	0.096	0.118

### AUV Attitude Decoupling Control Structure Design

Position control is the basis and key of the whole underwater navigation control of AUV (Sun et al., 2020c; Tao et al., 2021; Liu et al., 2022d). Its position control accuracy is determined by the attitude control accuracy, and the attitude control error of AUV will amplify its position control error, so in order to ensure the high accuracy control of speed and position during underwater navigation, its attitude must be accurately controlled. The Active Disturbance Rejection Control technique used in this paper enables accurate control of AUV attitude even in the complexity and variability of underwater disturbances. The system equations of the AUV are shown in Eq. 11. It can be shown that there are six variables that need to be controlled, while the output of attitude control is only three items, and coupling phenomenon exists in all three attitude angle directions. Therefore, in the following we propose a decoupled control method to control the attitude of the AUV.

$$\left[\begin{array}{l} F_{1}^{'}\\ F_{2}^{'}\\ F_{3}^{'}\\ F_{4}^{'}\\ F_{5}^{'}\\ F_{6}^{'} \end{array}\right]=\left[\begin{array}{c} F_{0}+F_{\varphi }+F_{\theta }\\ F_{0}+F_{\varphi }-F_{\theta }\\ F_{0}-F_{\varphi }-F_{\theta }\\ F_{0}-F_{\varphi }+F_{\theta }\\ 0-F_{\psi }\\ 0+F_{\psi } \end{array}\right]$$ (12)

Where the $F_{0}$ is the thrust of the 6 thrusters when the AUV is stationary in the water to maintain the desired state $F_{\varphi }$ , $F_{\theta }$ , $F_{\psi }$ ,, are respectively the adjustment forces in three directions φ , θ , ψ .

Thus the attitude decoupling control system of the AUV can be expressed as:

$$\left[\begin{array}{l} \overset{..}{\varphi }\\ \overset{..}{\theta }\\ \overset{..}{\psi } \end{array}\right]=\left[\begin{array}{c} \frac{I_{y}-I_{z}}{I_{x}}\dot{\theta }\dot{\psi }+\frac{4F_{\varphi }X}{I_{x}}\\ \frac{I_{z}-I_{x}}{I_{y}}\dot{\varphi }\dot{\psi }+\frac{4F_{\theta }Y}{I_{y}}\\ \frac{2F_{\psi }Y}{I_{z}} \end{array}\right]$$ (13)

### AUV Attitude ADRC Control Algorithm

**FIGURE 2:**
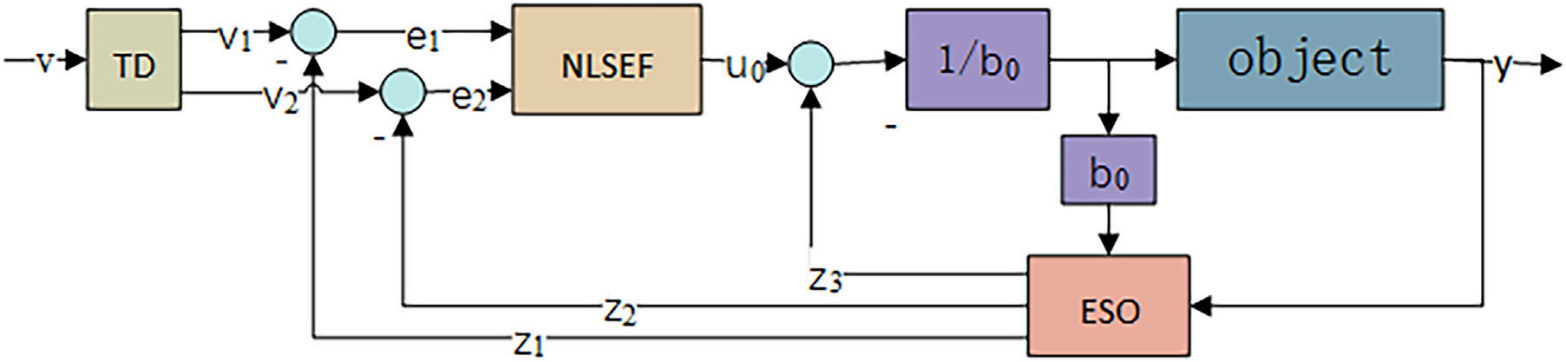
ADRC controller structure.

**FIGURE 3:**
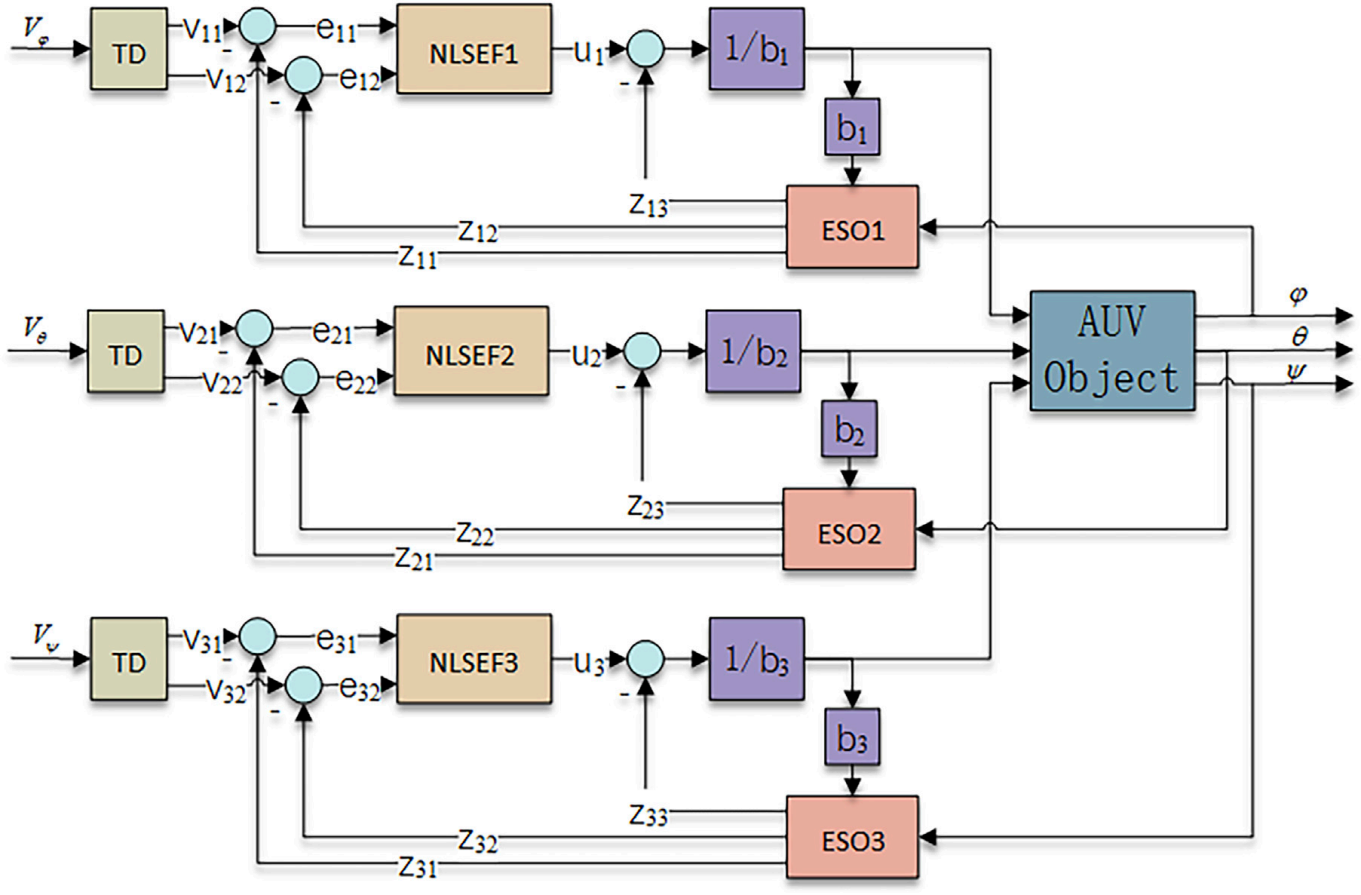
ADRC attitude control structure diagram.

ADRC has excellent immunity to perturbations and is not dependent on a specific system model. The ADRC consists of a tracking differentiator (TD), a nonlinear state error feedback control law (NLSEF), and an ESO. The literature (Wu et al., 2018; Luo et al., 2020) used ADRC to achieve decoupled control of MIMO nonlinear system; the literature (Fareh et al., 2019; Cheng et al., 2020) used ADRC to achieve control of flexible robotic arm; the literature (Qiao et al., 2020; Yu et al., 2020) used ADRC to achieve adaptive control of missile attitude; the structure of ADRC is shown in Figure 2:

From Eq. 13, it can be seen that the AUV attitude control system is a nonlinear system. In this paper, TD is used to give the transition to the input signal, ESO is used to realize the observation of the internal and external disturbance of the system, and the real-time compensation of the disturbance is realized in the NLSEF. Its corresponding ADRC attitude control structure is shown in Figure 3.

#### AUV Attitude Input Signal Tracking Process

For the input signal tracking problem of the second-order system, Prof. Jingqing Han derived a fastest integrated function fhan (Eq. 14), where d, a_0_, y, a_1_, a_2_, s_y_, a, s_a_ are intermediate quantities for the purpose of simplifying the equation, and this Function can achieve fast tracking of the input signal. The differential tracker of AUV attitude input signal is constructed by using this function as shown in Eq. 15

$$\left\{ \begin{array}{l} d=r_{0}h_{0}^{2}\\ a_{0}=h_{0}x_{2}\\ y=x_{1}+a_{0}\\ a_{1}=\sqrt{d\left(d+8\left|y\right|\right)}\\ a_{2}=a_{0}+\text{sign}\left(y\right)\left(a_{1}-d\right)/2\\ s_{y}=\left[\text{sign}\left(y+d\right)-\text{sign}\left(y-d\right)\right]/2\\ a=\left(a_{0}+y-a_{2}\right)s_{y}+a_{2}\\ s_{a}=\left[\text{sign}\left(a+d\right)-\text{sign}\left(a-d\right)\right]/2\\ \text{fhan}=-r\left[a/d-\text{sign}\left(\text{a}\right)\right]s_{a}-r_{0}\text{sign}\left(\text{a}\right) \end{array}\right.$$ (14)

$$\left\{ \begin{array}{l} x_{1}\left(k+1\right)=x_{1}\left(k\right)+hx_{2}\left(k\right)\\ x_{2}\left(k+1\right)=x_{2}\left(k\right)+h\text{fh}\\ \text{fh}=\text{fhan}\left(x_{1}\left(k\right)-v\left(k\right),x_{2}\left(k\right),r_{0},h_{0}\right) \end{array}\right.$$ (15)

$x_{1}$ is the tracking of the AUV attitude input signal v, $x_{2}$ is the tracking of the differential signal of v, r_0_ is the velocity factor, h_0_ is the filtering factor, and f_h_ is the intermediate quantity of the simplified equation.

#### ESO-Based AUV Perturbation Estimation

From Eq. 13, the pitch angle equation of state of the AUV can be written in the following form.

$$\left\{ \begin{array}{l} \dot{x_{1}}=x_{2}\\ \dot{x_{2}}=f\left(x_{1},x_{2},w\left(t\right),t\right)+bu_{\theta }\\ y=x_{1} \end{array}\right.$$ (16)

where $x_{1}=\theta$ ; $x_{2}=\dot{\theta }$ ; $b=\frac{4X}{I_{x}}$ ; w(t) are external disturbances; $u_{\theta }=F_{\theta }$ is the control quantity; and $f\left(x_{1},x_{2},w\left(t\right),t\right)$ is the complex disturbance.

Make $x_{3}=f\left(x_{1},x_{2},w\left(t\right),t\right)$ extend a new state that can turn the original system into a linear system by:

$$\left\{ \begin{array}{l} \dot{x_{1}}=x_{2}\\ \dot{x_{2}}=x_{3}+bu_{\theta }\\ \dot{x_{3}}=\dot{f}\left(x_{1},x_{2},w\left(t\right),t\right)=w_{0}\left(t\right)\\ y=x_{1} \end{array}\right.$$ (17)

For the above system the following observer is designed for observation.

$$\left\{ \begin{array}{l} \varepsilon_{1}=z_{1}-y\\ \dot{z_{1}}=z_{2}-\beta_{01}\varepsilon_{1}\\ \dot{z_{2}}=z_{3}-\beta_{02}\text{fal}\left(\varepsilon_{1},\frac{1}{2},\delta \right)+bu_{\theta }\\ \dot{z_{3}}=-\beta_{03}\text{fal}\left(\varepsilon_{1},\frac{1}{4},\delta \right) \end{array}\right.$$ (18)

The rectification parameters enable precise tracking of system state variables and accurate estimation of external disturbances. The heading and yaw angles of the AUV are also processed using this algorithm.

#### Nonlinear Error Feedback and Control Volume Generation for AUV

Based on the above observed disturbance and the tracking amount of the input signal, high precision control can be achieved by using the ADRC nonlinear error combination. The nonlinear error combination is shown as follows.

$$\left\{ \begin{array}{l} e_{11}=v_{11}-z_{11},e_{12}=v_{12}-z_{12}\\ u_{01}=\text{fhan}\left(e_{11},ce_{12},r,h_{1}\right)\\ e_{21}=v_{21}-z_{21},e_{22}=v_{22}-z_{22}\\ u_{02}=\text{fhan}\left(e_{21},ce_{22},r,h_{1}\right)\\ e_{31}=v_{31}-z_{31},e_{32}=v_{32}-z_{32}\\ u_{03}=\text{fhan}\left(e_{31},ce_{32},r,h_{1}\right) \end{array}\right.$$ (19)

The results of the nonlinear error feedback can be used to generate high-precision control quantities for the AUV.

$$\left\{ \begin{array}{l} u_{1}=\left(u_{01}-z_{13}\right)/b_{1}\\ u_{2}=\left(u_{02}-z_{23}\right)/b_{2}\\ u_{3}=\left(u_{03}-z_{33}\right)/b_{3} \end{array}\right.$$ (20)

where $u_{1}$ , $u_{2}$ , $u_{3}$ , respectively denote the control inputs of pitch angle, cross-roll angle, and heading angle,; b is the compensation factor.

### PSO Optimization of ADRC Control Parameters

ADRC needs to adjust more parameters, although most of them have reference values and have little impact on the system, however there are still damping factor c , accuracy factor $h_{1}$ , compensation factor $b_{0}$ that need to be adjusted, and the adjustment process is complicated. The Particle Swarm Optimization algorithm (PSO) is one of the evolutionary algorithms (Lalwani et al., 2019; Sun et al., 2020d; Zhao et al., 2022). The solution process of PSO starts from a set of random initial solutions and iterates continuously to find the optimal solution through the neighborhood search computational method, in each iteration of the computation, the particle swarm updates itself through the individual optimal solution and the population optimal solution until it finds the optimal solution. Compared to evolutionary algorithms such as genetic algorithms, Particle Swarm Optimization have no crossover and mutation operations, can be encoded with real numbers, have a simple structure and are fast to calculate, which have great advantages as algorithms for online optimization (Chegini et al., 2018; Tian et al., 2020; Liao et al., 2021). In this paper, we use PSO to offline optimize three parameters that have a large impact on ADRC: the damping factor c , the accuracy factor $h_{1}$ , and the compensation factor $b_{0}$ .

#### ADRC Control Adaptation Function

According to the performance requirements of AUV control, the steady-state error, response time, and overshoot are taken into account in the evaluation index, and the constructed fitness function is shown in Eq. 21. $e_{ss}$ , $e_{t}$ , and $M_{p}$ represent the steady-state error, response time, and overshoot, and $\eta_{i}$ (i = 1,2,3) is the corresponding weights.

$$J=\int_{0}^{t}\left(\eta_{1}\left|e_{ss}\right|\right)\text{d}t+\eta_{2}e_{t}+\eta_{3}M_{p}$$ (21)

#### PSO Optimized ADRC Controller Flow

**FIGURE 4:**
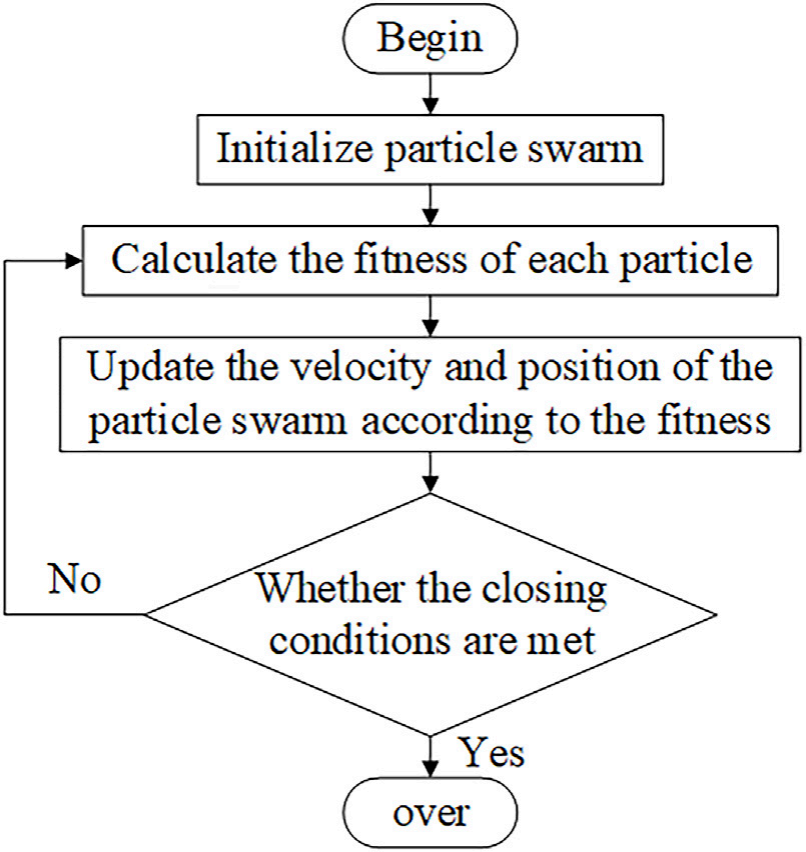
Flow chart of particle swarm optimization.

Step 1: Initialize the particle swarm and ADRC parameters. Set the random position and velocity of the particle swarm, and calculate the local optimal and global optimal initial values of the particle swarm.
Step 2: The position vector of each particle is used as the three parameters of the ADRC controller: damping factor, accuracy factor, and compensation factor. The adaptation value of each particle is calculated according to Eq. 21.
Step 3: Compare the adaptation value of each particle with the local optimum and the global optimum, and update the position and velocity of the particle swarm.
Step 4: Determine whether the end condition is reached. If not, return to Step 2, otherwise stop the optimization and output the global optimum parameters. Figure 4 shows the flow chart of the PSO algorithm.

## Simulation Experiments

**FIGURE 5:**
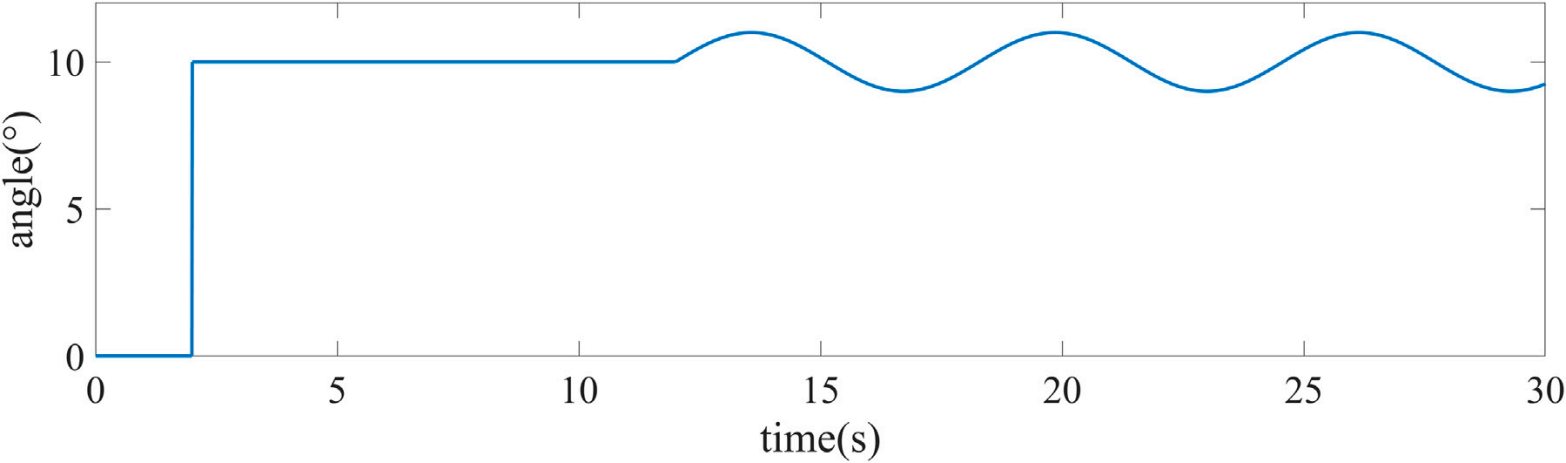
Tracking the target signal.

**FIGURE 6:**
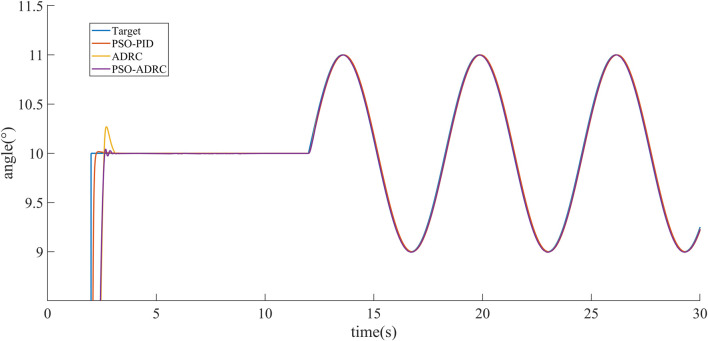
Signal tracking under no interference.

Simulation experiments are performed for the above control objects. In order to reflect the control effect of particle swarm optimized ADRC, it is compared with Particle Swarm Optimized PID algorithm (Girirajkumar et al., 2010) and conventional ADRC. From *AUV Mathematical Model*, it can be shown that the three attitude angle control equations of AUV are basically the same, so it is only shown that the effect of the simulation control of pitch angle in this paper. Figure 5 shows the tracking target signal for the pitch angle, where the 2nd second is step signals and the 12th second starts with a sinusoidal signal. The simulation of PSO-PID, ADRC and PSO-ADRC in the absence of interference is shown in Figure 6. The response speed and overshoot of PSO-PID in the absence of disturbances are better than those of ADRC and PSO-ADRC, and the overshoot of ADRC optimized by particle swarm is smaller than that of ADRC.

**FIGURE 7:**
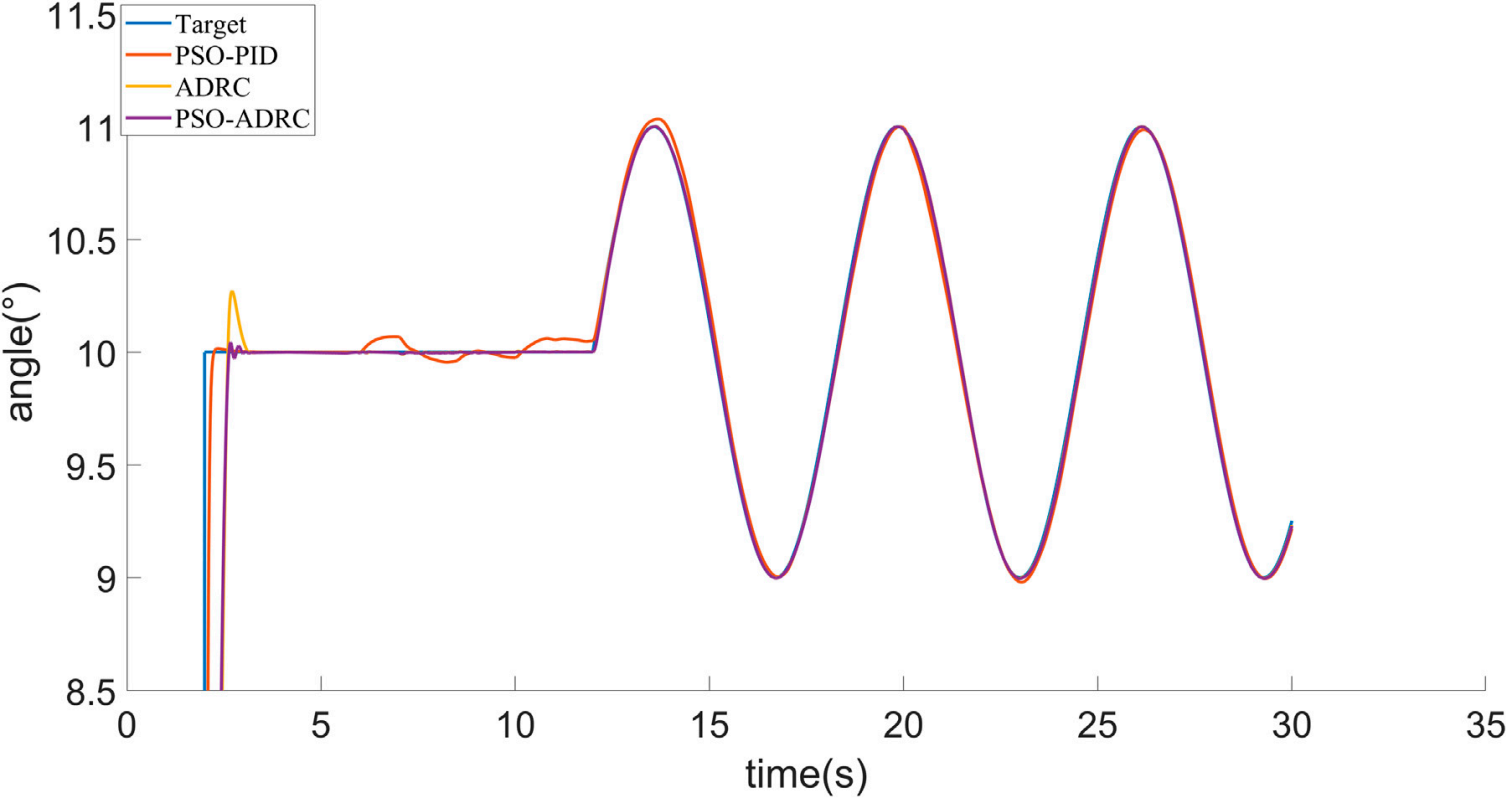
Signal tracking experiment under external interference.

**FIGURE 8:**
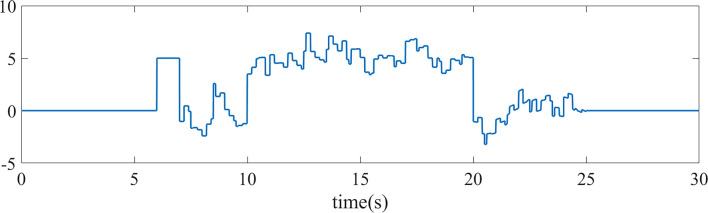
External interference signal.

**FIGURE 9:**
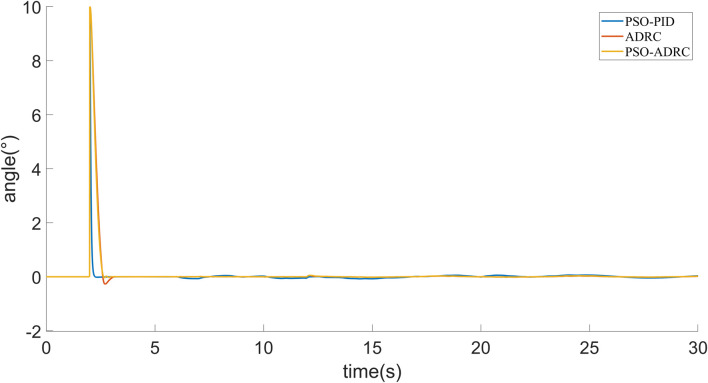
Tracking error under external disturbance.

Figure 7 shows the tracking effect of the signal under external disturbance, and Figure 8 shows the random external disturbance. The PSO-PID controller shows significant perturbations under the influence of external disturbances, while the control effect is basically unaffected by the two ADRCs due to the mechanism of observing and compensating the perturbations based on ESO, and the signal tracking error under external disturbances is smaller than that of the PSO-PID algorithm (Figure 9, Table 2).

**TABLE 2:** Tracking effect with external interference.

	Response time/s	Overshoot/%	Steady-state error/%
PSO-PID	2.242	0.2	0.7
ADRC	2.601	2.7	0.1
PSO-ADRC	2.628	0.4	0.07

**FIGURE 10:**
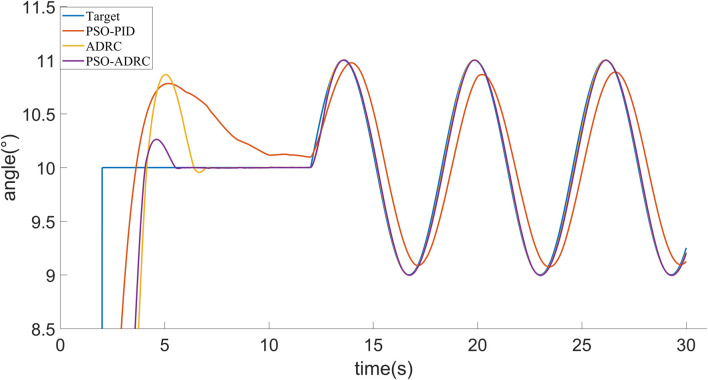
Signal tracking experiment under internal interference.

**FIGURE 11:**
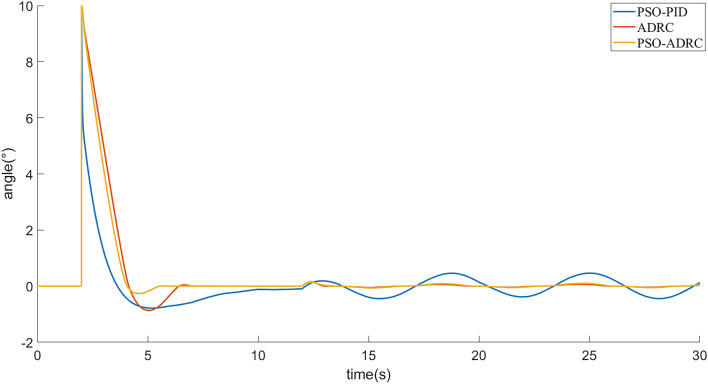
Tracking error under internal disturbance.

Figure 10 shows the simulation experiment of changing the internal parameters of the system, by adding the internal disturbance. The response speed of three controllers is reduced and the overshoot is increased. The effect of PSO-PID controller is the most affected and basically fails to satisfy the control requirements, while the effect of ADRC controller optimized by using PSO in this paper is the least affected (Figure 11, Table 3).

**TABLE 3:** Tracking effect with internal interference.

	Response time/s	Overshoot/%	Steady-state error/%
PSO-PID	3.627	7.8	1.15
ADRC	4.141	8.7	0.04
PSO-ADRC	4.063	2.6	0.02

From the appeal simulation experiments, it can be shown that PSO-ADRC has strong robustness to interference, which has better generality at the same time. After the parameters are optimized by PSO, the parameters of actual control object has changed within a certain range, which does not affect the effect of the controller. These characteristics of PSO-ADRC have strong applicability for AUV working in complex and changing underwater environment.

## Conclusion

The research on AUV attitude control based on decoupling algorithm and PSO-ADRC is motivated by the fact that it can facilitate the stable control of AUV attitude and improve the operational capability of AUV. In this paper, a decoupling algorithm for a six-degree-of-freedom AUV is proposed to solve the problem of serious coupling in each variable and to realize that one output variable of attitude angle corresponds to one input variable. The problem with current AUV control methods is that immunity and versatility cannot be combined, however the ADRC controller can guarantee AUV immunity while not relying on an accurate mathematical model of the AUV. The parameters of the ADRC optimized by the PSO algorithm can greatly reduce the process of manually adjusting the parameters.

## Data Availability

The original contributions presented in the study are included in the article/Supplementary Material, further inquiries can be directed to the corresponding authors.
